# An Enhanced K-Means Algorithm for Water Quality Analysis of The Haihe River in China

**DOI:** 10.3390/ijerph121114400

**Published:** 2015-11-12

**Authors:** Hui Zou, Zhihong Zou, Xiaojing Wang

**Affiliations:** 1 School of Economics and Management, Beihang University, Beijing 100191, China; E-Mails: huizou@cau.edu.cn (H.Z.); star_wxj@163.com (X.W.); 2 School of Science, China Agricultural University, Beijing 100083, China

**Keywords:** water classification, indicator weight, local optimization

## Abstract

The increase and the complexity of data caused by the uncertain environment is today’s reality. In order to identify water quality effectively and reliably, this paper presents a modified fast clustering algorithm for water quality analysis. The algorithm has adopted a varying weights K-means cluster algorithm to analyze water monitoring data. The varying weights scheme was the best weighting indicator selected by a modified indicator weight self-adjustment algorithm based on K-means, which is named MIWAS-K-means. The new clustering algorithm avoids the margin of the iteration not being calculated in some cases. With the fast clustering analysis, we can identify the quality of water samples. The algorithm is applied in water quality analysis of the Haihe River (China) data obtained by the monitoring network over a period of eight years (2006–2013) with four indicators at seven different sites (2078 samples). Both the theoretical and simulated results demonstrate that the algorithm is efficient and reliable for water quality analysis of the Haihe River. In addition, the algorithm can be applied to more complex data matrices with high dimensionality.

## 1. Introduction

The evaluation of water quality is essentially a classification problem [1]. Due to the fact that current water quality assessment standards are not uniform, research on unsupervised methods is quite active. There are two common methods of unsupervised classification, namely, cluster analysis (CA), specially hierarchical cluster analysis (HCA), and principal component analysis (PCA). These methods have been widely used in water quality management [2,3,4,5,6], but owing to the increase and the complexity of data in the water environment, water quality evaluation using these methods faces much pressure in data handling. Currently, the key technologies for large data analysis are K-means clustering, fuzzy C-means clustering, fuzzy logic, evolutionary algorithms, and so forth [7,8]. In K-means clustering, the Euclidean distances with equal weights method is widely used [8,9,10]. Recently, some research has focused on using the Euclidean distance with varying weights. In fact, in the field of water quality, there are several means to determine weights. The variance of each indicator can stand for the weight of the indicator [11]. Weights have been calculated by the superscale, which is the ratio of the value of every indicator at each monitoring point over the corresponding water quality standard [12,13]. Considering the difficulty of the fuzzy synthetic evaluation method in calculation of the multiple factors and the lack of knowledge about the relationship among evaluated objects, a new weight evaluation process using an entropy method was introduced [14]. The F statistics of water samples was normalized as the weight of fuzzy comprehensive evaluation for determining the source of water inrush in a coal mine [15]. Based on use of the membership functions and coefficient of variation as the weights, four fuzzy similarity measures were used to classify water samples of the Haihe River into the proper water quality standard ranks [16]. Indicator weighting can be considered as the generalization of indicator selection since it assigns a proper weight value to each indicator instead of giving either one, to retained indicators, or zero, to eliminated indicators [17]. In other words, the objective of indicator selection is also achieved by selecting the indicators that have higher weights from the indicator weighting process. In short, the purpose of indicator weighting for clustering is to assign proper weight values for all indicators according to their importance in the clustering quality. Although a great deal of research about the selection of weights has been done, there are few studies in the field on searching the local optimization for the weights. Improved K-means clustering algorithms, by an elegant and natural generalization of Fishers discriminant analysis to select the best indicator weighting, have been proposed [18,19,20].

In this study, a modified indicator weight self-adjustment algorithm based on K-means was used, incorporating the classification of the water quality via searching for local optimization of the weights, whereby the quality of clustering was improved. Then all monitoring data were classified into reasonable ranks.

## 2. Materials and Methods

### 2.1. Dataset

**Figure 1:**
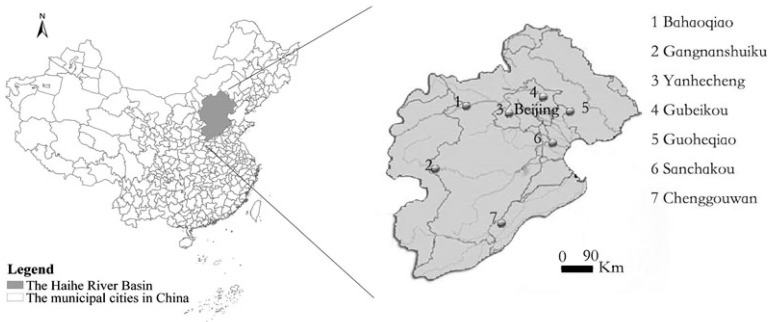
Location of the Heihe River in China and location of the monitoring stations.

The Haihe River is the biggest river system in North China and includes all rivers flowing into the Bohai Sea. The east coastline of the watershed extends from Shanhaiguan to the old Yellow River estuary, and the total area of the watershed is about 318,200 km^2^. The main stream runs through Hebei Province, Beijing City, Tianjin City and Shandong Province. The location of the river in China and the location of the monitoring stations are illustrated in Figure 1. The dataset from seven water quality monitoring stations on the Haihe River (Yanhecheng, Gubeikou, Gangnanshuiku, Guoheqiao, Sanchakou, Bahaoqiao and Chenggouwan), comprising four water quality indicators monitored weekly over eight years (2006–2013), was obtained from the Ministry of Environmental Protection of China. There were 2078 samples in all after eliminating unreasonable data and data worse than grade V. Samples in which one of the indicators exceeded the standard of grade V (*i.e.*, grade VI) were not included in the analysis because most data worse than grade V were far from the boundaries and could be considered as outliers from a statistical point of view and would affect cluster quality. The available water quality indicators included pH, dissolved oxygen (DO), chemical oxygen demand (COD) and ammonia nitrogen (NH_3_-N). The surface water environmental quality standards (GB3838-2002) for DO, COD and NH_3_-N are listed in Table 1. The boundary values of DO, COD and NH_3_-N defined in Table 1 and the sample mean of pH were defined as original K cluster centroid. The descriptive statistics are summarized in Table 2. There are five grades in GB3838-2002 omitting grade VI.

**Table 1:** Boundary values of some indicators in the GB3838-2002 water quality standard.

Indicator	I	II	III	IV	V
DO (mg/L)	7.5	6	5	3	2
COD (mg/L)	2	4	6	10	15
NH_3_-N (mg/L)	0.15	0.5	1	1.5	2

**Table 2:** Descriptive statistics of water quality indicators.

Indicator	Mean	SD	SE	Minimum	Maximum
pH	8.07	0.43	0.01	6.34	9.35
DO (mg/L)	9.02	2.83	0.06	2.02	25.5
COD (mg/L)	3.51	2.40	0.05	0.2	15
NH_3_-N (mg/L)	0.40	0.44	0.01	0.01	2

### 2.2. Dataset Treatment

In the Knowledge Discovery in Databases (KDD) process, data cleaning and preprocessing is an important step before choosing the data mining algorithms and data mining. Data cleaning and preprocessing includes basic operations, such as deciding on strategies for appropriately handling missing data fields, removing noise or outliers [21].

For missing data, ignoring the tuple is usually done when the class label is missing. It is not effective when the percentage of missing values per attribute varies considerably [22]. In this case there were only 18 missing tuples, so they were ignored.

In all normal distributions, the range μ ± 3σ includes nearly all cases, where μ denotes mean and σ denotes standard deviation. After z-score normalization, values higher than 3 or lower than −3 are outliers and they were deleted [22].

Most multivariate statistical methods require variables to conform to the normal distribution, thus, the normality of the distribution of each indicator was checked by analyzing kurtosis and skewness index before multivariate statistical analysis. In all cases, the variable distribution was far from normal [11,23]. The original data demonstrated that kurtosis values range from 0.268 to 25.118 and skewness value range from −0.343 to 3.985, indicating that the variable distribution was far from normal with 95% confidence. Since most of kurtosis and skewness values were far from zero, the original data were transformed in the form $x^{\prime }=\log_{10}（x）$ [4,23]. After log- transformation, the kurtosis and skewness values ranged from −2.380 to 0.092 and 0.025 to 14.893, respectively. In the case of CA, all log-transformed variables were also z-scale standardized (the mean and variance were set to zero and one, respectively) to minimize the effects of different units and variance of variables and to render the data dimensionless [3,24].

### 2.3. Modified Indicator Weight Self-Adjustment K-Means Algorithm (MIWAS-K-Means)

Clustering is a fundamental technique of unsupervised learning in statistics and machine learning [25]. Clustering is generally used to find groups of similar items in a set of unlabeled data. How to select the best indicator weighting is a crucial question. Let $X=\left\{x_{1},\cdots x_{m},\cdots x_{M}\right\}$ be a data set with M data objects and $F=\left\{f_{1},\cdots f_{n},\cdots f_{N}\right\}$ be an indicator set with *N* indicators. A sample of X can be represented as a data object $x_{m}=\left\{x_{m1},\cdots x_{mn},\cdots x_{mN}\right\}$ . The K-means algorithm partitions X into K clusters. Let $C=\left\{C_{1},\cdots C_{k},\cdots C_{K}\right\}$ be a set of K clusters, coupled with a set of corresponding cluster center $c_{k}=\left\{c_{k1},\cdots c_{k2},\cdots c_{kN}\right\},\;k=1\cdots K.$ In addition, $\left\|C_{k}\right\|$ means the number of data objects to $C_{k}$ such that $\sum_{k=1}^{K}\left\|C_{k}\right\|=M.$ $c_{kn}(k=1\cdots K,n=1\cdots N)$ is defined as $c_{kn}=\sum_{i=1}^{\left\|C_{k}\right\|}x_{h_{i}n}/\left\|C_{k}\right\|,\;C_{k}=\left\{x_{h_{1}}\cdots x_{h_{i}}\cdots x_{h_{\;\left\|C_{k}\right\|}}\right\},$ k=1⋯K, n=1⋯N. Let $g=(g_{1},\cdots ,g_{n},\cdots ,g_{N})$ be the global center of all M data objects in the dataset, where $g_{n}$ is defined as $g_{n}=\sum_{m=1}^{M}x_{mn}/M.$

Taking indicator weight into account, let $W=\left\{w:\sum_{n=1}^{N}w_{n}=1,w_{n}\geq 0,1\leq n\leq N\right\}$ be a data set of all possible indicator weights. The weight of an indicator should reflect the importance of the indicator to cluster quality. Note that each indicator weighting leads to a different partitioning of the dataset. Intuitively, we would like to minimize the separations within clusters and maximizing the separations between clusters. Hence, the objective function is (Equation (1)):

(1) $$\begin{array}{cc} MaximizeV(\hat{U},\hat{c},w,\hat{g})=\frac{S^{\prime }(\hat{c},w,\hat{g})}{S(\hat{U},\hat{c},w)} & =\frac{\sum_{k=1}^{K}\left(\left\|C_{k}\right\|\times \sum_{n=1}^{N}w_{n}\times d(c_{kn},g_{n})\right)}{\sum_{m=1}^{M}\sum_{k=1}^{K}\sum_{n=1}^{N}\left(u_{mk}\times w_{n}\times d(x_{mn},c_{kn})\right)}\\ & =\frac{\sum_{n=1}^{N}\left[w_{n}\times \left(\sum_{k=1}^{K}\left\|C_{k}\right\|\times d(c_{kn},g_{n})\right)\right]}{\sum_{n=1}^{N}\left[w_{n}\times \left(\sum_{m=1}^{M}\sum_{k=1}^{K}u_{mk}\times d(x_{mn},c_{kn})\right)\right]}\\ \mathrm{Subject to}\left\{ \begin{array}{l} \sum_{n=1}^{N}w_{n}=1\\ w_{n}\geq 0,n=1,2,\cdots ,N \end{array}\right. & \end{array}$$

where $u_{mk}\in \left\{0,1\right\},\;\sum_{k=1}^{K}u_{mk}=1,\;0<\sum_{m=1}^{M}u_{mk}<m,$ $u_{mk}\in \left\{0,1\right\}$ denotes the membership degree of the *m-th* sample belonging to the *K-th* cluster. $S(\hat{U},\hat{c},w)$ is the sum of all separations within clusters and $S^{\prime }(\hat{c},w,\hat{g})$ is the sum of all separations between clusters. $d(x_{mn},c_{kn})={\left|x_{mn}-c_{kn}\right|}^{2}$ is the difference between $x_{m}$ and $c_{k}$ in terms of the *n-th* feature $f_{n}$ and $d(c_{kn},g_{n})={\left|c_{kn}-g_{n}\right|}^{2}$ is the difference between $c_{k}$ and g in terms of the *n-th* feature $f_{n}$ .

Set $a_{n}=\sum_{m=1}^{M}\sum_{k=1}^{K}u_{mk}\times d(x_{mn},c_{kn})$ , $b_{n}=\sum_{k=1}^{K}\left\|C_{k}\right\|\times d(c_{kn},g_{n})$ , where $a_{n}$ represents the sum of separations within clusters in terms of the *n-th* indicator and $b_{n}$ represents the sum of separations between clusters in terms of the *n-th* indicator. Hence, Equation (1) can be rewritten as:

(2) MaximizeV(U^,c^,w,g^)=∑n=1Nwn×bn∑n=1Nwn×an  Subject to {∑n=1Nwn=1wn≥0,n=1,2,⋯,N

The model given by Equation (2) is a linear programming problem and its feasible solution is located at the corner points of the convex polygon bounded by the N+1 linear constraints in Equation (2) [26]. By taking the corner points into Equation (2), the objective values will respective be $\frac{b_{1}}{a_{1}},\cdots \frac{b_{n}}{a_{n}},\cdots \frac{b_{N}}{a_{N}}$ . The maxization of Equation (2) can occur at the corner-point $\frac{b_{l}}{a_{l}}$ when $\frac{b_{l}}{a_{l}}\geq \frac{b_{n}}{a_{n}}\;$ for  n=1,2,⋯N(l≠n). Accordingly, indicator weights in W are specified as (Equation (3)):

(3) {wn=1,  if  bl/al<bn/an;wn=0,  otherwise.

There are two philosophies behind the classification method. One is that each indicator contributes to the water quality classification. Meanwhile there is another philosophy that states that if one indicator exceeds the standard of a certain grade, the water immediately loses its functions belonging to lower grades. If for drinking water one parameter exceeds the standard, it is not suitable for drinking water any more, regardless the value for the other parameters. If for reclaimed water, it is suitable to use after disposal. From these options, we follow the first philosophy. Therefore, there is an unreasonable situation that the winner-take-all phenomenon makes other indicator weights becomes insignificant, even though they may contribute a lot to the cluster quality. A weight-adjusting procedure is combined with the original K-means algorithm. By increasing the weight of the indicator $w_{l}$ having a higher $b_{l}/a_{l}$ value, the indicator weights are adjusted [19]. The method is as following:

Let $W^{\left(s\right)}=\left\{w^{\left(s\right)}:\sum_{n=1}^{N}{w_{n}}^{\left(s\right)}=1,{w_{n}}^{\left(s\right)}\geq 0,1\leq n\leq N\right\}$ be the set of the N indicator weights at the *s-th* iteration and each indicator weight at the *(s+1)-th* iteration can be adjusted by adding an adjustment margin $\Delta w_{n}^{\left(s\right)}$ at the s-th iteration as Equation (4):

(4) $${w_{n}}^{\left(s+1\right)}={w_{n}}^{\left(s\right)}+\Delta w_{n}^{\left(s\right)},\;n=1,2,\cdots ,N$$

Considering the contribution of the indicator to clustering quality, the adjustment margin $\Delta w_{n}^{\left(s\right)}$ can be derived according to its ${b_{n}}^{\left(s\right)}/{a_{n}}^{\left(s\right)}$ value at the *s-th* iteration as Equation (5):

(5) $$\Delta w_{n}^{\left(s\right)}=\frac{{b_{n}}^{\left(s\right)}/{a_{n}}^{\left(s\right)}}{\sum_{n=1}^{N}{b_{n}}^{\left(s\right)}/{a_{n}}^{\left(s\right)}},\;n=1,2,\cdots ,N$$

Note that the adjusted weight in (4) needs to be normalized to a value between 0 and 1. Through the normalization, each adjusted indicator weight ${w_{n}}^{\left(s+1\right)}$ can be derived as (Equation (6)):

(6) $${w_{n}}^{\left(s+1\right)}=\frac{1}{2}\left({w_{n}}^{\left(s\right)}+\Delta w_{n}^{\left(s\right)}\right),\;n=1,2,\cdots ,N$$

There is a shortcoming of this algorithm that $a_{n}^{\left(s\right)}$ is perhaps equal to zero if all samples in a cluster have the same values or do not occur on an indicator, which causes $\Delta w_{n}^{\left(s\right)}$ to not be calculated. To avoid the problem, an improved algorithm is proposed which introduces a constant σ to change the adjustment margin as Equation (5), in order to avoid the difficulty in the computation [20] (Equation (7)):

(7) $$\Delta w_{n}^{\left(s\right)}=\frac{{b_{n}}^{\left(s\right)}/\left({a_{n}}^{\left(s\right)}+\sigma \right)}{\sum_{n=1}^{N}\left[{b_{n}}^{\left(s\right)}/\left({a_{n}}^{\left(s\right)}+\sigma \right)\right]},\;n=1,2,\cdots ,N$$

where σ is the average dispersion of the entire data set for all indicators.

We note that it is an approximate method and the definition in Equation (1) is unreasonable. In this paper, we will propose an improved algorithm to avoid the shortcoming. Note that $\sum_{k=1}^{K}\left(\left\|C_{k}\right\|\times \sum_{n=1}^{N}w_{n}\times d(c_{kn},g_{n})\right)$ in Equation (1) represents the separations between clusters. $g=(g_{1},\cdots ,g_{n},\cdots ,g_{N})$ is the global center of all M data objects in the dataset. We think that the definition is unreasonable and modify it as (Equation (8)):

(8) $$\sum_{k=1}^{K}\left(\left\|C_{k}\right\|\times \sum_{j=1}^{K}\sum_{n=1}^{N}w_{n}\times d(x_{kn},c_{jn})\right)\;k,j=1\cdots K,k\neq j.$$

where $c_{jn}(j=1\cdots K,n=1\cdots N)$ is defined as (Equation (9)):

(9) $$c_{jn}=\sum_{i=1}^{\left\|C_{j}\right\|}x_{l_{i}n}/\left\|C_{j}\right\|,\;C_{j}=\left\{x_{l_{1}}\cdots x_{l_{i}}\cdots x_{l_{\;\left\|C_{j}\right\|}}\right\},j=1\cdots K,n=1\cdots N.$$

Hence, the objective function is (Equation (10)):

(10) $$\begin{array}{ll} MaximizeV(\hat{U},\hat{c},w,x)=\frac{S^{\prime }(\hat{c},w,x)}{S(\hat{U},\hat{c},w)} & =\frac{\sum_{k=1}^{K}\left(\left\|C_{k}\right\|\times \sum_{j=1}^{K}\sum_{n=1}^{N}w_{n}\times d(x_{kn},c_{jn})\right)\;}{\sum_{m=1}^{M}\sum_{k=1}^{K}\sum_{n=1}^{N}\left(u_{mk}\times w_{n}\times d(x_{mn},c_{kn})\right)}\\ & =\frac{\sum_{n=1}^{N}\left[w_{n}\times \left(\sum_{k=1}^{K}\sum_{j=1}^{K}\left\|C_{k}\right\|\times d(x_{kn},c_{jn})\right)\right]}{\sum_{n=1}^{N}\left[w_{n}\times \left(\sum_{m=1}^{M}\sum_{k=1}^{K}u_{mk}\times d(x_{mn},c_{kn})\right)\right]} \end{array}$$

$$\text{Subject to }\left\{ \begin{array}{l} \sum_{n=1}^{N}w_{n}=1\\ w_{n}\geq 0,n=1,2,\cdots ,N \end{array}\right.$$

$S(\hat{U},\hat{c},w)$ is the sum of all separations within clusters and $S^{\prime }(\hat{c},w,x)$ is the sum of all separations between clusters. Set $a_{n}=\sum_{m=1}^{M}\sum_{k=1}^{K}u_{mk}\times d(x_{mn},c_{kn}),\;b_{n}=\sum_{k=1}^{K}\sum_{j=1}^{K}\left\|C_{k}\right\|\times d(x_{kn},c_{jn}).\;a_{n}$ represents the sum of separations within clusters in terms of the *n-th* indicator and $b_{n}$ represents the sum of separations between clusters in terms of the *n-th* indicator. Hence, Equation (10) can be rewritten as (Equation (11)):

$$\begin{array}{l} MaximizeV(\hat{U},\hat{c},w,\hat{g})=\frac{\sum_{n=1}^{N}w_{n}\times b_{n}}{\sum_{n=1}^{N}w_{n}\times a_{n}} \end{array}$$

(11) $$\text{Subject to}\{\begin{array}{l} \sum_{n=1}^{N}w_{n}=1\\ w_{n}\geq 0,n=1,2,\cdots ,N \end{array}$$

Accordingly, indicator weights in *w* are specified as (Equation (12)):

(12) $$\left\{ \begin{array}{l} w_{n}=1,\;b_{l}/a_{l}<b_{n}/a_{n},\;l=1,2\cdots ,N\;and\;l\neq n;\\ w_{n}=0,\;\mathrm{otherwise}. \end{array}\right.$$

Note that if $b_{l}/a_{l}<b_{n}/a_{n},\;0\leq b_{l},a_{l},b_{n},a_{n}\leq 1$ , then $b_{l}-a_{l}\leq b_{n}-a_{n}$ . In fact, $b_{l},a_{l},b_{n},a_{n}$ is not in the interval [0,1]. Therefore, a simple normalization is used as following (Equation (13)):

(13) $$a_{n}^{0}=\frac{a_{n}}{\sum_{n=1}^{N}a_{n}};b_{n}^{0}=\frac{b_{n}}{\sum_{n=1}^{N}b_{n}}\text{, }n=1,2,\cdots N.$$

Each indicator weight at the *(s+1)-th* iteration can be adjusted by adding an adjustment margin $\Delta w_{n}^{\left(s\right)}$ at the *s-th* iteration as Equation (14):

(14) $${w_{n}}^{\left(s+1\right)}={w_{n}}^{\left(s\right)}+\Delta w_{n}^{\left(s\right)},\;n=1,2,\cdots ,N$$

The adjustment margin $\Delta w_{n}^{\left(s\right)}$ can be derived according to its ${b_{n}^{0}}^{\left(s\right)}/{a_{n}^{0}}^{\left(s\right)}$ value at the *s-th* iteration as Equation (15):

(15) $$\Delta w_{n}^{\left(s\right)}=\frac{{b_{n}^{0}}^{\left(s\right)}-{a_{n}^{0}}^{\left(s\right)}}{\sum_{n=1}^{N}\left({b_{n}^{0}}^{\left(s\right)}-{a_{n}^{0}}^{\left(s\right)}\right)},\;n=1,2,\cdots ,N$$

Therefore, each adjusted indicator weight ${w_{n}}^{\left(s+1\right)}$ can be derived as (Equation (16)):

$${w_{n}}^{\left(s+1\right)}=\frac{{w_{n}}^{\left(s\right)}+\frac{{b_{n}^{0}}^{\left(s\right)}-{a_{n}^{0}}^{\left(s\right)}}{\sum_{n=1}^{N}\left({b_{n}^{0}}^{\left(s\right)}-{a_{n}^{0}}^{\left(s\right)}\right)}}{\sum_{n=1}^{N}{w_{n}}^{\left(s\right)}+\sum_{n=1}^{N}\frac{{b_{n}^{0}}^{\left(s\right)}-{a_{n}^{0}}^{\left(s\right)}}{\sum_{n=1}^{N}\left({b_{n}^{0}}^{\left(s\right)}-{a_{n}^{0}}^{\left(s\right)}\right)}}$$

(16) $$\;=\frac{1}{\left(1+1\right)}\left({w_{n}}^{\left(s\right)}+\frac{{b_{n}^{0}}^{\left(s\right)}-{a_{n}^{0}}^{\left(s\right)}}{\sum_{n=1}^{N}\left({b_{n}^{0}}^{\left(s\right)}-{a_{n}^{0}}^{\left(s\right)}\right)}\right)=\frac{1}{2}\left({w_{n}}^{\left(s\right)}+\Delta w_{n}^{\left(s\right)}\right)\text{, }n=1,2,\cdots ,N.$$

In the whole clustering process, if any parameter was not set, the improved algorithm above updates the indicator weights by the accurate adjustment margin and avoids $\Delta w_{n}^{\left(s\right)}$ not being calculated.

**Figure 2:**
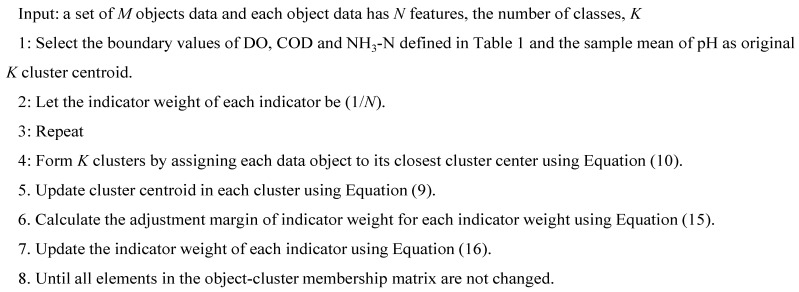
The pseudo-code for the MIWAS-K-means algorithm

The pseudo-code of modified indicator weight self-adjustment K-means algorithm named MIWAS-K-means is illustrated in Figure 2, the number of classes is selected as five according to GB3838-2002. The MIWAS-K-means algorithm repeats the assignment, update, and weight adjustment procedures until all elements in the object-cluster membership matrix are not changed.

## 3. Results and Discussion

### 3.1. Evaluation Measures

Various cluster validity measures can be used to evaluate the performance of a clustering algorithm [27]. When the class labels of experimental data are unknown, unsupervised measures are used for the evaluation task. A typical and popular unsupervised measure is the Sum of Square within-cluster Error (SSE), shown as Equation (17):

(17) $$Minimise\;S(U,c,w)=\sum_{m=1}^{M}\sum_{k=1}^{K}\sum_{n=1}^{N}\left(u_{mk}\times w_{n}\times d(x_{mn},c_{kn})\right)$$

subject to:

$$\left\{ \begin{array}{l} \sum_{k=1}^{K}u_{mk}=1\\ u_{mk}\in \{1,0\}\\ \sum_{n=1}^{N}w_{n}=1\\ w_{n}\geq 0. \end{array}\right.\;for\;m=1,2,\ldots ,M;\;k=1,2,\ldots ,KN;\;n=1,2,\ldots ,N$$

SSE is especially important because the real world clustering applications seldom reveal information about the class labels of data. The lower the SSE value that an algorithm generates, the better the clustering quality of the algorithm is.

The clustering performances for K-means and MIWAS-K-means are shown in Table 3. We note that the two algorithms use the same initial cluster centers. Based on the SSE measure, the performance of the proposed MIWAS-K-means is obviously superior to the K-means. However, as indicated in Table 3, the MIWAS-K-means algorithm needs more iterations to achieve computational convergence as compared to the K-means algorithm.

**Table 3:** The performance for the two clustering algorithms.

Clustering Algorithms	K-Means	MIWAS-K-Means
SSE	899.6053	782.2792
Number of iterations	12	18
Final feature weights	(0.25,0.25,0.25,0. 25)	(0.1602,0.1978, 0.5116,0.1303)

### 3.2. Weights of Features

The MIWAS-K-means algorithm was applied to classify the water quality data of Haihe River from seven sites using 2078 samples collected from 2006 to 2013. Weights of indicators were determined through minimizing the separations within cluster and maximizing the separations between clusters. The weight of each indicator is shown in Table 4. From this table we can see that the weight of COD was the greatest among all four, so it was the most significant index that affected water quality. Number of times COD was in a higher grade than the other water quality indicators is 1486.

**Table 4:** Weights of indicators calculated by improved weighted K-means algorithm.

Indicators	pH	DO	COD	NH_3_-N
Weights	0.1602	0.1978	0.5116	0.1303

In the Haihe River basin, the discharge of COD is mainly from the urban domestic sewage discharges and the industrial wastewater discharges. In 2005, the total population of the river basin was 0.13 billion, or 9.7% of the national population. The gross domestic product (GDP) of the basin area was 2575 billion RMB Yuan, representing approximately 14.1% of the national value. Although Beijing and Tianjin are developed regions, most of the cities in the basin are less developed regions. In conjunction with rapid population and economic growth, the basin has suffered severe water deterioration from both point and nonpoint source pollution. Meanwhile, the Haihe River basin is an important industrial base and high technology industry base in China, where the main industries are metallurgy, power, chemical, machinery, electronics, coal. In addition high technology industries, such as electronic information, biotechnology, new energy and new materials, have been developing rapidly. The proportion of high water consumption industry and heavy pollution industry in the whole river basin is still large.

In the Water Pollution Prevention and Control Planning Report (2011–2015), China’s water bodies were divided into five classes according to their pollution status, and the basin was classified as the most polluted class. According to the Haihe River Basin Water Resources Bulletins announced by the Haihe Water Conservancy Commission (HWCC), in recent decades COD, total nitrogen (TN), and total phosphorous (TP) were the dominant water pollution indicators in the area based on the water quality reports from the basin.

Furthermore, the weights of the parameters indicated a few significant parameters with high weights and non-essential parameters with low weights responsible for water quality classification. Therefore, the weights could determine the important parameters and reduce the number of sampling parameters, especially in large data set. It was essential to strengthen the monitoring accuracy of the few significant parameters which is useful to the optimization of regional water quality monitoring network. For instance, the 5-year COD reduction percentage is an essential control variable in the point source amount control system. In the Twelfth Five Year Plan for key river basins in China, the mandated goal for COD emission reduction every 5 years is 9.7%.

### 3.3. Water Quality Classification

Based on the weights obtained above, the 2078 samples were clustered into five clusters and the water quality level determined. Descriptive means of water quality indicators in five clusters and numbers of samples are listed in Table 5. Cluster 1 represents 502 records with the lowest values of COD and NH_3_-N and highest values of DO. Values of COD and NH_3_-N become higher and higher while value of DO becomes lower and lower from cluster 1 to cluster 3. The mean COD in cluster 4 was higher than the value in cluster 5, while the means of NH_3_-N in cluster 5 was higher than the value in cluster 5. It could be inferred that samples in cluster 4 were mainly influenced by COD emissions, while samples in cluster 4 are mainly influenced by emissions of NH_3_-N.

**Table 5:** Mean values of water quality features and numbers of cases in five clusters.

	Cl.1	Cl.2	Cl.3	Cl.4	Cl.5
pH	7.89 ± 0.39	8.12 ± 0.40	8.12 ± 0.44	8.3 ± 0.35	7.7 ± 0.43
DO	9.43 ± 1.99	9.65 ± 2.40	8.75 ± 2.44	8.49 ± 2.99	3.97 ± 1.61
COD	1.45 ± 0.33	2.38 ± 0.37	4.71 + 0.11	8.95 ± 2.25	7.20 ± 2.46
NH_3_-N	0.17 ± 0.22	0.24 ± 0.28	0.54 ± 0.53	0.92 ± 0.85	1.37 ± 1.10
Number of cases	502	700	545	194	137

### 3.4. Verifying of Classification Accuracy

Cross-validation is important in guarding against testing hypotheses suggested by the data called Type III errors [28]. It is a generally applicable way to predict the performance of a model on a validation set with computation in mathematical analysis.

Leave-one-out cross-validation (LOOCV) involves using a single observation from the original sample as the validation data and the remaining observations as the training data. This process is repeated until each observation in the sample is used once as the validation data.

LOOCV was applied to the data clustered by MIWAS-K-means algorithm in order to verify the accuracy of classification. Assignment percentages are shown in Table 6. We find that all the correct assignment percentages are more than 94.9%. Therefore it was inferred that the majority of the samples obtained an appropriate label.

**Table 6:** Correct and wrong assignments obtained by LOOCV.

	1	2	3	4	5
1	97.4	2.6	0	0	0
2	3.4	94.6	0	2	0
3	0	0	96.9	2.1	1
4	0	5	1.1	92.8	1.1
5	0	0	2.9	3.6	93.4

### 3.5. Analysis of the Pollution Sources

Table 7 demonstrates the mean values with standard deviation of water quality indicators at the seven sites. The mean of DO is relatively higher and the value of COD and NH_3_-N is relatively lower of Yanhecheng, Gubeikou, Gangnanshuiku, Guoheqiao. The mean of DO is relatively lower and the value of COD and NH_3_-N is relatively higher in Sanchakou and Bahaoqiao. The mean of DO is lowest while the value of COD and NH_3_-N is highest in Chenggouwan.

**Table 7:** Mean values with standard deviation of water quality indicators in 7 sites.

	pH	DO	COD	NH_3_-N
Yanhecheng	8.22 ± 0.47	8.97 ± 1.94	3.31 ± 1.38	0.24 ± 0.18
Gubeikou	7.91 ± 0.41	8.64 ± 1.79	2.10 ± 1.09	0.19 ± 0.10
Gangnanshuiku	7.91 ± 0.29	9.74 ± 1.45	1.75 ± 0.30	0.07 ± 0.04
Guoheqiao	8.18 ± 0.39	10.3 ± 3.04	2.56 ± 0.81	0.31 ± 0.17
Sanchakou	8.19 ± 0.45	8.66 ± 3.7	6.95 ± 2.65	0.83 ± 0.70
Bahaoqiao	7.88 ± 0.40	7.30 ± 1.81	4.54 ± 1.39	1.11 ± 0.79
Chenggouwan	8.15 ± 0.46	4.22 ± 3.33	10.1 ± 3.38	1.92 ± 1.52

Table 8 shows the number of observations in each cluster of the seven monitoring sites. Samples of Gangnanshuiku are all classified into cluster 1 and cluster 2. The majority of samples in Gubeikou is classified into cluster 1 and cluster 2. Most of the observations in Yanhecheng and Guoheqiao are classified into clusters 2 and 3. Most of observations in Bahaoqiao are classified into cluster 3. Furthermore, most of the observations from Sanchakou and Chenggouwan are classified into clusters 4 and 5.

**Table 8:** Number of observations in each cluster of the seven monitoring sites.

	Sum	Cl.1	Cl.2	Cl.3	Cl.4	Cl.5
Yanhecheng	353	32	150	155	14	2
Gubeikou	372	160	166	42	2	2
Gangnanshuiku	354	238	116	0	0	0
Guoheqiao	392	68	240	82	1	1
Sanchakou	326	0	9	102	143	72
Bahaoqiao	238	4	19	163	23	29
Chenggouwan	43	0	0	1	11	31

Therefore, we classified the seven sampling sites into four groups (A, B, C, and D). Group A consisted of Gangnanshuiku and Gubeikou. Group B consisted of Yanhecheng and Guoheqiao. Group C consisted of Bahaoqiao. Group D consisted of Sanchakou and Chenggouwan. In group A, Gubeikou and Gangnanshuiku were located near the Miyun reservoir and Gangnan reservoir, respectively. The two reservoirs are the major drinking water headwater sites of the capital Beijing and the provincial capital of Shijiazhuang. They were relatively far from pollution sources and had a better protection of water resources. In group B, Yanhecheng was located at the exit of the Guanting reservoir near Beijing. The water quality was improved by improving water resource protection measures and the mixing dilution effect of the reservoir water. Guoheqiao was located near the entrance to the Yuqiao reservoir. The upstream is the streamway which is leading the clean Luan river into Tianjin. The site in group C was located near the entrance to the Guanting reservoir which is close to pollution sources. Zhangjiakou is located in the upper reaches of the reservoir and is an industrial base in North China, with more than 10 thousand large manufacturing sites. Industrial effluent without appropriate treatment is directly discharged into the river. In addition, the slow flow caused by a large bend at the entrance leads to precipitation of pollutants at the site. Sites in group D are all located near unsewered areas, and there are many small paper mills and breweries along the river, therefore the water quality was easily influenced by wastewater from agricultural irrigation and household upstream and industrial effluent.

## 4. Conclusions

In this paper, a modified varying weights K-means cluster algorithm is proposed to classify the water quality in the Haihe River in China. The new algorithm avoids the margin of the iteration not being calculated in some cases and improves the efficiency of data processing. Simulation results show that the algorithm can efficiently and reliably analyze the discrimination of water quality in the Haihe River and determine the most significant indexes that affect water quality. It improves the efficiency of data processing in Haihe River water quality testing, and provides a reliable scientific basis for water pollution control in the Haihe River. The algorithm can be applied not only to large data analysis and processing, but also provides some guidance for others area in the large data processing field.
